# Strain-level typing and identification of bacteria – a novel approach for SERS active plasmonic nanostructures

**DOI:** 10.1007/s00216-018-1153-0

**Published:** 2018-06-16

**Authors:** Evelin Witkowska, Dorota Korsak, Aneta Kowalska, Anna Janeczek, Agnieszka Kamińska

**Affiliations:** 1 Institute of Physical Chemistry, Polish Academy of Sciences, Kasprzaka 44/52, 01-224 Warsaw, Poland; 2 Faculty of Biology, Institute of Microbiology, Department of Applied Microbiology, University of Warsaw, Miecznikowa 1, 02-096 Warsaw, Poland

**Keywords:** *Listeria monocytogenes*, SERS, Bacteria detection, Bacteria identification, Strain level discrimination, *bcrABC*, BcrB, BcrC, CadA1, CadA2, PCA

## Abstract

One of the potential applications of surface-enhanced Raman spectroscopy (SERS) is the detection of biological compounds and microorganisms. Here we demonstrate that SERS coupled with principal component analysis (PCA) serves as a perfect method for determining the taxonomic affiliation of bacteria at the strain level. We demonstrate for the first time that it is possible to distinguish different genoserogroups within a single species, *Listeria monocytogenes,* which is one of the most virulent foodborne pathogens and in some cases contact with which may be fatal. We also postulate that it is possible to detect additional proteins in the *L. monocytogenes* cell envelope, which provide resistance to benzalkonium chloride and cadmium. A better understanding of this infectious agent could help in selecting the appropriate pharmaceutical product for enhanced treatment.

**Electronic supplementary material:**

The online version of this article (10.1007/s00216-018-1153-0) contains supplementary material, which is available to authorized users.

**Graphical abstract:**
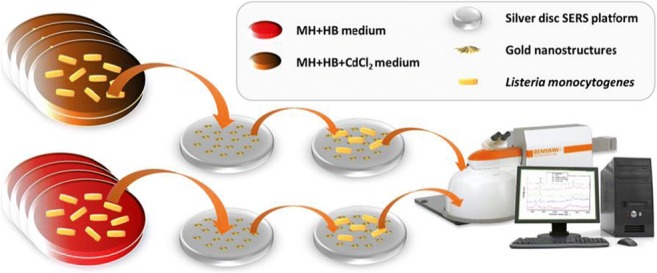
ᅟ

## Introduction

Surface-enhanced Raman scattering (SERS) is a technique in which a Raman signal is amplified by several orders of magnitude because of the electromagnetic interaction of light with metal nanostructures [1, 2]. This technique may be applied in numerous fields of science and life, for instance in the detection and identification of chemical compounds, e.g., poisons, narcotic substances [3], or biological substances, e.g., DNA [4], viruses [5], cancer cells [6]. SERS also provides an opportunity to obtain a spectral fingerprint of different bacterial species, including foodborne species, for instance *Listeria monocytogenes* [7–9]. For this reason, over the last decade interest in SERS applied to bacteria has rapidly increased [10–12]. This interest is associated with the high sensitivity of the SERS technique and increasing number of bacterial strains dangerous for humans. Moreover, the SERS method enables the detection of even a single bacterium in an analyzed sample [13], making the method more reliable.

Current SERS methods of bacteria detection are performed mainly at the species level. Such experiments are important, e.g., in the case of the detection of *Salmonella* spp., *Cronobacter* spp. or *Listeria monocytogenes* in food samples. These bacteria are listed in Commission Regulation (EC) No. 2073/2005 on microbiological criteria for foodstuffs and were successfully detected via the SERS method from different food matrices [14]. It was also proven that SERS discrimination of two bacterial species belonging to one genus (*L. monocytogenes* and *L. ivanovii*) is possible [14]. However, bacteria identification at the strain level is also an important issue as some strains, even within the same species, may be very virulent and pathogenic for humans while others are not. Differentiation between these two groups, pathogenic and non-pathogenic, is crucial in clinical microbiology and in medical and pharmaceutical treatment. Strain level identification is important not only in the matter of determining whether a patient should be prescribed an antibiotic, but also in terms of selecting the appropriate drug (e.g., when a specific bacterial strain is resistant to a particular antibiotic). One of the first SERS-based bacteria identification at this level was carried out by Walter et al. [15], who showed that SERS spectra of various *Escherichia coli* strains differ in the intensity of two bands located at 731 and 1331 cm^-1^. The identification of different strains belonging to single bacterial species was performed also for *Vibrio parahemolyticus* [16]. Additionally, it was shown that SERS-based strain level identification is suitable for differentiation of *E. coli*, *Klebsiella pneumoniae*, *Staphylococcus saprophyticus*, and *Enterococcus faecalis* because of the differences in their antibiotic susceptibility profile [17]. In this paper, we demonstrate that SERS coupled with PCA (principal component analysis) is also suitable for discrimination of *L. monocytogenes –* it allows the identification of *L. monocytogenes* at the genoserotype level and the detection of some membrane proteins within a single *L. monocytogens* genoserogroup.

*L. monocytogenes*, one out of 17 species qualified to *Listeria* genus, is an important foodborne pathogen [18] that can cause serious human infections, such as bacteremia and central nervous system infections, primarily in neonates and immunocompromised adults. It can also cause perinatal infections that may result e.g., in abortions [19]. This organism is one of the most important causes of death from foodborne infections in industrialized countries [20, 21]. Human disease cases and outbreaks caused by this organism have a considerable economic impact on society and the food industry [22]. The main route of infection is through the ingestion of contaminated food products [23], as *L. monocytogenes* can persist in food processing environments [24–26].

In this study, we demonstrate the possibility of distinguishing *L. monocytogenes* bacteria at strain level by the SERS method. More specifically, we prove that it is possible to differentiate *L. monocytogenes* strains belonging to: (1) different genoserogroups, and (2) a single genoserogroup. The differences in the first case are based on the variance in cell surface antigens, and in the second case, on the presence of additional proteins in the bacterial cell envelope, e.g., CadA1, CadA2 (proteins determining resistance to Cd^2+^, encoded by *cadA1* and *cadA2* genes, respectively), BcrB and BcrC (proteins determining resistance to benzalkonium chloride, encoded by *bcrABC* gene). These changes are expressed in the SERS spectrum. Further development of this method may serve clinical laboratories to define whether the analyzed bacterium is able to contaminate food samples/disinfectants and thus whether it is dangerous for humans.

## Materials and methods

### Preparation of the Ag-Au bimetallic SERS substrate

The Ag-Au bimetallic substrate was synthesized according to slightly modified, previously published procedures [27].

In order to obtain the Ag-Au SERS substrates, silver discs (Ø = 10 mm, H = 5 mm) were mechanically polished with Al_2_O_3_ slurries, first with the particle size of 0.5 mm, and second, with 0.3 mm. After polishing, the silver discs were rinsed with 70% C_2_H_5_OH solution. Then, in order to remove the adsorbed Al_2_O_3_ particles and other possible contaminations, the discs were sonicated for 10 min in 70% C_2_H_5_OH solution. Subsequently, the Ag discs were rinsed with Millipore water and sonicated for 10 min in Millipore water. The sonication process was then repeated with a new portion of Millipore water. Next, the discs were electrochemically roughened (oxidation/reduction cycles, ORC) in the electrochemical cell filled with 0.1 M KCl solution. The three ORCs were applied (0.5 V and –0.5, both for 40 s; 0.5 V and –0.5, both for 15 s; 0.5 V for 15 s and –0.5 for 30 s). In the end the reduction potential of –0.4 V was applied for 300 s. After electrochemical roughening the discs were washed in Millipore water and dried.

In order to deposit gold nanostructures onto the roughened surface of the silver discs, the electrochemical cell was first filled with a solution of 0.4 mM HAuCl_4_ in 0.1 M HClO_4_ and then the potential of 5 mV was applied for 200 s. Next, the Ag-Au bimetallic SERS substrates obtained as described above were rinsed with Millipore water, dried, and placed in desiccator.

### Raman spectroscopy and surface-enhanced raman spectroscopy

In the SERS measurements we used Ag-Au bimetallic SERS substrates onto which the bacterial samples were placed. In order to perform SERS measurements, a Renishaw inVia Raman system equipped with a diode laser with wavelength of 785 nm was used. The laser light was passed through a line filter and focused on a sample mounted on an X–Y–Z translation stage with a 50× microscope objective (Leica, NA = 0.25). The microscope was equipped with 1200 grooves per mm grating, cutoff optical filters, and a 1024 × 256 pixel Peltier-cooled RenCam CCD detector, which allowed registering the Stokes part of Raman spectra with 5–6 cm^-1^ spectral resolution and 2 cm^-1^ wavenumber accuracy. The laser beam diameter, defined as twice the radius of the circle encompassing the area with 86% of the total power, was about 2.3 μm; approximately the same values were obtained from an experimentally obtained laser spot image and from the theoretical formula (4λf/πD). The experiments were performed under ambient conditions using a back-scattering geometry of 0.5 mW power at the sample.

The SERS spectra were recorded immediately after placing the analyzed sample onto the SERS substrate. All bacteria were at the same growth stage at the time of sample preparation. The SERS spectra were collected from 25 different points for each sample in mapping mode (20 × 40 μm). The recording of SERS spectra was completed after ~30 min from placing the sample on the Ag-Au substrate. The spectra were processed with the Wire3 software provided by Renishaw.

### Bacterial strains

*L. monocytogenes* strains: 475/05, 43/04, 06/09/s, 2082/03, 06/09, 02/07, 82/04, 16/09, and 01/07 were obtained from the Department of Applied Microbiology, University of Warsaw, Poland.

The samples were collected from large retail outlets, smaller units, and food-producing factories over a 7 y period – from 2004 to 2010. Samples were collected in five cities in central and north-east areas of Poland. All food samples were transported to the laboratories inside portable insulated cold boxes, whereas the swabs were moved in sterile tubes. The samples were immediately subjected to microbiological analysis, which was carried out in accredited laboratories. *L. monocytogenes* isolates recovered from different types of food and food-related sources (pork neck, broccoli, cold cuts, smoked salmon, dumplings, raw salad, smoked herring, smoked blue warehou fillets).

The isolates were serotyped by multiplex PCR, according to the procedure ‘*in-house method: Listeria monocytogenes molecular serotyping’* described by the European Union Reference Laboratory for *L. monocytogenes* ANSES (Maison-Alfort, France) using primers Lmo0737-1 and Lmo0731, Lmo1118-1 and Lmo1118-2, ORF2110-1 and ORF2110-2, ORF2819-1 and ORF2819-2, PRS1 and PRS2, LIP1, and LIP2. The second PCR assay was performed to detect the presence of the *fla*A gene (primers flaA-F and flaA-R) [28–30]. Differentiation of *L. monocytogenes* isolates based on PCR reaction is shown on Figs. S1 and S2 (see Electronic Supplementary Material, ESM).

Identification of the two different *cadA* determinants involved the use of the primers cadA-Tn5422F and cadA-Tn5422R for *cadA1* gene, associated with transposon Tn5422, the primers cadA-pLM80F and cadA-pLM80R for *cadA2* gene harbored on plasmid pLM80 [31]. Primers p1 and p2 were used to produce a PCR fragment containing *bcrABC* cassette [32]. The names and sequences of the discussed primers used for PCR are listed in Table S1 in the ESM.

BC (benzalkonium chloride) and cadmium (Cd^2+^) susceptibility of *L. monocytogenes* isolates is a mechanism connected with the resistance of *L. monocytogenes* bacteria to benzalkonium chloride and cadmium, respectively. It was assessed as described previously by Mullapudi et al., 2008 [33]. Strains were considered resistant to BC, cadmium if they yielded confluent growth on Mueller Hinton agar with 5% horse blood (MH+HB; Oxoid) supplemented with 10 μg/mL of benzalkonium chloride (Sigma Aldrich), 75 μg/mL cadmium chloride (Sigma Aldrich) [33].

The group affiliation of each strain and the presence of *bcrABC* cassette, *cadA1* and *cadA2* genes is shown in Table 1.

**Table 1:** The group affiliation of *L. monocytogenes* strains used in experiments

*Strain*	*Source of isolation*	Genoserogroup*	*bcrABC*	*cadA1*	*cadA2*	BC^**^	Cd^2+***^
475/05	Pork neck	gr IIa	–	–	–	□	□
43/04	Broccoli	gr IIa	–	+	–	□	■
06/09/S	Cold cuts	gr IIa	+	–	+	■	■
2082/03	Smoked salmon	gr IIc	–	–	–	□	□
06/09	Dumplings	gr IIc	–	–	+	□	■
02/07	Smoked blue warehou	gr IVb	–	–	–	□	□
82/04	Raw salad	gr IVb	–	+	–	□	■
16/09	Dumplings	gr IVb	–	–	+	□	■
01/07	Smoked herring	gr IVb	+	–	+	■	■

(+) present; (–) absent

^*^gr IIa - (serotypes 1/2a-3a), gr IIc (serotypes 1/2c-3c), and IVb (serotypes 4b-4d-4e);

^**^BC - benzalkonium chloride;

^***^Cd^2+^ - cadmium cation;

□ - sensitive (growth inhibited on MH+HB medium supplemented with 10 μg/ml benzalkonium chloride and/or 75 μg/ml cadmium chloride);

■ - resistant (confluent growth on MH+HB medium supplemented with 10 μg/ml benzalkonium chloride and/or 75 μg/mL cadmium chloride)

### Sample preparation

**Fig. 1:**
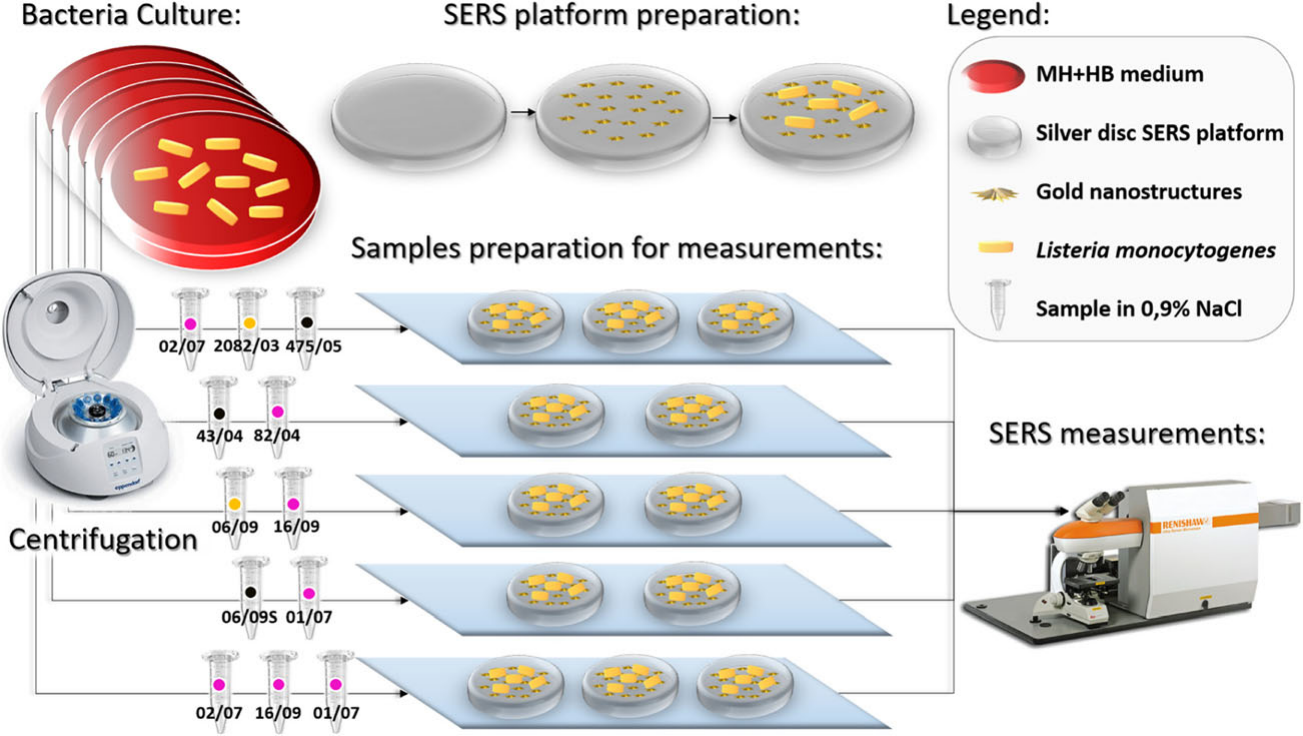
Simplified scheme of the experiment, part I. The color of the dot on each sample indicates a specific genoserogroup: black- group IIa, yellow- group IIc, and pink- group IVb

**Fig. 2:**
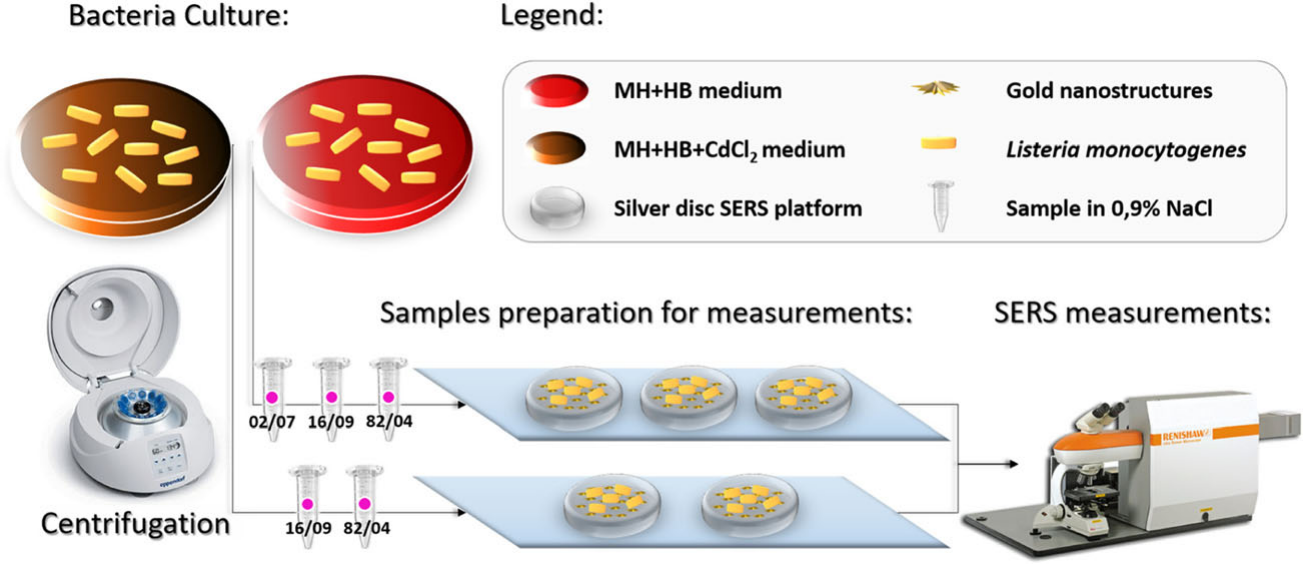
Simplified scheme of the experiment, part II. The pink color of the dot on each sample indicates the genoserogroup IVb

The bacterial isolates were cultured on MH+HB agar and/or MH+HB supplemented with 75 μg/mL anhydrous cadmium chloride in order to be multiplied. The plates were incubated at 37 °C for 24 h. After incubation, three single colonies were placed via sterile plastic inoculating loop into 50 μL of sterile 0.9% NaCl solution, mixed and centrifuged for 5 min at 4000 rpm. The centrifugation process in the saline solution was repeated three times to obtain a solution of pure bacterial cells. The bacteria were finally dispersed in 10 μL of sterile 0.9% NaCl solution. The mixtures were subsequently placed onto the SERS substrates and measured after ~5 min with Raman spectrometer. The concept of the entire experiment is presented in Fig. 1 and in Fig. 2.

In order to estimate the amount of colony forming units in one analyzed sample, three bacterial colonies of similar diameters of ~1 mm were suspended in 1 mL of saline solution. For this purpose, a serial 10-fold dilution of bacterial suspension was performed. The volume of 100 μL of bacterial suspension was plated and after incubation in 37 °C for 24 h the bacterial colonies were counted. The obtained concentration of bacterial cells was 5.7 × 10^9^/mL.

### PCA - spectral data analysis

The SERS spectra were prepared for principal component analysis (PCA) using a two-step approach. First, using a built-in OPUS software (Bruker Optic GmbH 2012 version) the spectra were smoothed with Savitzky-Golay filter, the background was removed using baseline correction (concave rubber band correction; no. of iterations 10, no. of baseline points 64), and then the spectra were normalized using a Min-Max normalization. All the data were transferred to the Unscrambler@ software (CAMO software AS, ver. 10.3, Norway), where the PCA was performed.

### Data set used in PCA

In this study the analysis was performed over the entire spectral region between 500 and 1630 cm^-1^. For PCA, the following strains were compared (Fig. 1):

- three strains within three genoserogroups, all without *cadA1*, *cadA2*, and *bcrABC* genes (control groups): 02/07 (gr IVb), 2082/03 (gr IIc),and 475/05 (gr IIa)
- two strains within two genoserogroups both with *cadA1* gene: 43/04(gr IIa) and 82/04(gr IVb)
- two strains within two genoserogroups both with *cadA2* gene: 06/09 (gr IIc) and 16/09 (gr IVb)
- two strains within two genoserogroups both with *cadA2* and *bcrABC* genes: 06/09/S (gr IIa) and 01/07 (gr IVb)
- three strains within one genoserogroup: 02/07 (gr IVb) - control group, 16/09 (gr IVb) with *cadA2* gene, and 01/07 (gr IVb) with *cadA2* and *bcrABC* genes

Additionally, we compared, via PCA, strains within a single genoserogroup (Fig. 2):

- grown on MH+HB agar: 02/07 (gr IVb) - control group, 16/09 (gr IVb) with *cadA2* gene, and 82/04 (gr IVb) with *cadA1* gene
- grown on MH+HB supplemented with 75 μg/mL anhydrous cadmium chloride: 16/09 (gr IVb) with *cadA2* gene and 82/04 (gr IVb) with *cadA1* gene

This additional experiment was performed to check whether the *cadA* genes expression would be seen as a change of the SERS spectrum.

## Results and discussions

In this study, Ag-Au bimetallic substrates were used in all SERS experiments. As was shown in our previous work [27], these particular SERS-active substrates display a SERS EF of 10^7^, and thus demonstrate the ability to perform detection of low-concentration standard analytes like *p*-mercaptobenzoic acid or biological samples such as ABO antigens [34] or bacterial cells [27]. By applying the Ag-Au bimetallic substrate, we combined the characteristic features of both metals: high chemical stability of Au with very high Raman scattering enhancement for Ag. The SERS-active substrate used in this study exhibits, besides a uniformly high enhancement factor, high reproducibility and stability of recorded signals across a single substrate and between different substrates. The morphology of these SERS-active substrates was visualized by scanning electron microscopy (SEM) technique and is presented in Fig. S3 (see ESM). The SEM images of Ag-Au hybrid surfaces covered with individual bacteria cells are presented in Fig. S4A (see ESM).

Bacterial strains belonging to a single species of the *Listeria* genus differ in the antigenic determinants localized on the cell surface. These variances are the result of the presence of different chemical compounds that enter the structure of bacterial membrane proteins and extracellular organelles. Such dissimilarities between strains may be recognized by serologic typing, which allows for a more detailed analysis of *Listeria* spp. This typing is determined by the heat-stable somatic antigen O (15 subtypes: I-XV) and heat-labile ciliary antigen H (4 subtypes: A-D) [35]. Through examination of group-specific *Listeria* O and H antigens, 13 serotypes (i.e., 1/2a, 1/2b, 1/2c, 3a, 3b, 3c, 4a, 4b, 4c, 4d, 4e, 4ab, and 7) have been recognized in *L. monocytogenes* that differ in virulence potential [36]. It has been observed that at least 95% of *L. monocytogenes* strains isolated from food and 98% of clinical strains isolated from human listeriosis belong to serotypes 1/2a, 1/2b, 1/2c, and 4b and among these serotypes 1/2a is the most prevalent in food and 4b is the most frequently detected serotype from human listeriosis cases [37–39]. This observation shows how important the information about strain of specific bacterium is.

Serotyping has been widely used to characterize *L. monocytogenes* isolates and is important in epidemiological investigations. Rapid and practical molecular serogroup-related PCR typing of *L. monocytogenes* has been developed to differentiate the major *L. monocytogenes* serotypes. As a result, five distinct genoserogroups were distinguished: IIa (1/2a-3a), IIb (1/2b-3b-7), IIc (1/2c-3c), IVa (4a-4c), IVb (4ab-4b,4d-4e) [29, 30].

**Fig. 3:**
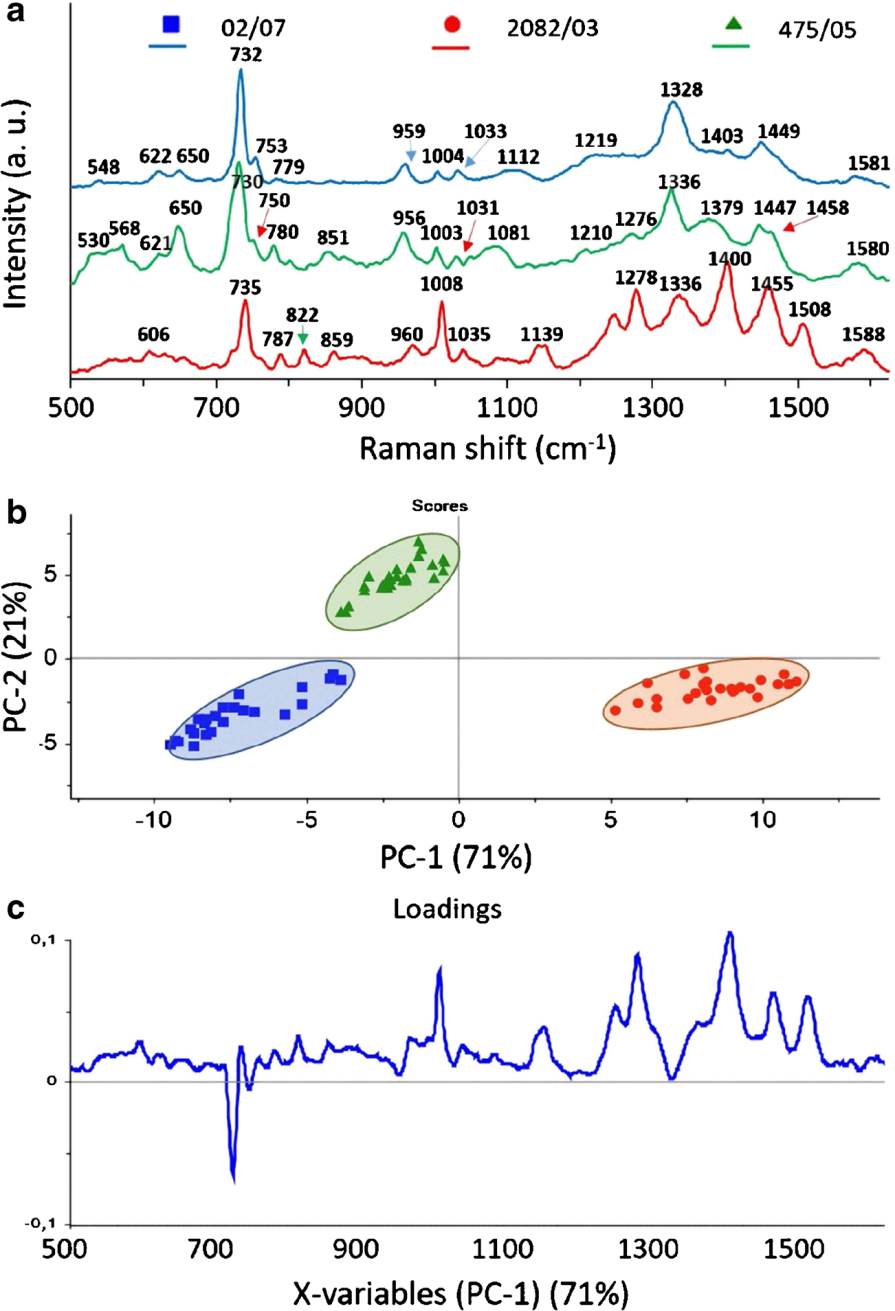
Average SERS spectra (**a**), PCA scores (**b**), and loadings (**c**) of three *L. monocytogenes* control strains (02/07, 2082/03, 475/05), all sensitive to BC and Cd^2+^, from three different genoserogroups, filtered from saline solution and recorded on Ag:Au SERS platforms. For all spectra the excitation wavelength was at 785 nm, laser power at 0.5 mW, and acquisition time 45 s. Each SERS spectrum was averaged from at least 25 measurements at different points of the SERS platform

In order to distinguish strains of *L. monocytogenes*, we first compared the spectra of three control strains, 475/05, 2082/03, and 02/07 from groups IIa, IIc, and IVb, respectively. As is clear in Figs. 3a, 5a, 6, and 7a, all gathered SERS spectra contain many similar features. In almost every spectrum, bands at ca. 621, 650, 730, 750, 780, 957, 1003, 1033, 1100, 1220, 1330, 1380, 1403, 1448, and 1580 cm^-1^ can be observed. The peaks at 730 cm^-1^ and 1330 cm^-1^ are assigned to the adenine part of flavin adenine dinucleotide (FAD). The band at ca. 650 cm^-1^ comes probably from tyrosine, those at 750 and 780 cm^-1^ from cytosine or uracil, at 1003 cm^-1^ from phenylalanine, at 957 cm^-1^ from C=C deformation or C-N stretching, and at 1033 cm^-1^ from C-C stretching in phospholipids. The peak at 1100 cm^-1^ can be assigned to C-O-C stretching in carbohydrates, the band at 1220 cm^-1^ to thymine, and at 1403 cm^-1^ to COO- symmetric stretching. The band at 1448 cm^-1^ can be linked to CH_2_ deformation and at 1580 cm^-1^ to ring stretching in adenine and phenylalanine. The band assignment was based on the research of Luna-Pineda et al., 2007 [40] and Demirel et al., 2009 [41]. Moreover, we measured the reproducibility of the SERS spectra of all analyzed strains of *L. monocytogenes* and present an example in Fig. S4B (see ESM) for strain 16/09. The presented spectra were recorded in mapping mode within 20 × 24 μm area of SERS-active substrate.

Although all *L. monocytogenes* strains give mainly the same peaks in SERS spectra, one can still observe differences. These dissimilarities are seen as changes in band intensities and shapes. In Fig. 3a, one can notice changes for all strains, e.g., (1) for the strain 02/07 the present band at about 1112 cm^-1^ and almost absent bands at ~ 780, 1380, and 1403 cm^-1^ in comparison with two other strains; (2) for the strain 2082/03 increased intensity in the 530–570 cm^-1^ region, as well changed band intensities at about 650, 960, 1080 cm^-1^ in comparison with two other strains; and (3) for strain 475/05 an additional band at 822 and 1508 cm^-1^, changes in the 1240–1270 cm^-1^ region, and increased intensity of peaks at 1400 and 1455 cm^-1^.

Owing to these changes, almost each strain exhibits a characteristic spectrum. The multivariate technique in the form of principal component analysis (PCA) was applied to the obtained SERS data in order to improve the sensitivity of differentiation. The PCA was performed onto preprocessed SERS spectra (smoothened, backgrounded, and normalized) in the entire recorded spectral region (500–1630 cm^-1^). Examination of the calculated scores and loadings for the most important PCs, as determined from percent variance plots, were used to investigate changes in the spectral features of SERS data. The two principal components, PC-1 and PC-2, were calculated, which together accounted for 92% of variability and allowed for the differentiation between all studied *L. monocytogenes* strains. A score plot (Fig. 3b) shows that the spectra could be divided into three groups (clusters) corresponding to 02/07 (blue), 2082/03 (red), and 475/05 (green) strains, from three different genoserogroups. Moreover, the loadings of the PCs provide information on the variables (wavenumber of the spectrum) that are important for group separation. Figure 3c displays the loadings plot of PC1 for the entire wavenumber region. By analyzing these plots, one can indicate the most important diagnostic variables in the analyzed data set. Variables with high loading values are the most important for diagnostic purposes. As can be seen, the wavenumber of 732 cm^-1^ has the largest weights in the opposite direction. There are other wavenumbers at ca. 1004, 1278, 1400, 1450, 1508 cm^-1^, which also have an important contribution to PC1 in the same direction. Taking into account the weight of each of these PC1 loadings, it is obvious that in particular five bands at 730, 1004, 1278, 1400, and 1450 cm^-1^ are the main contributors to PC1 and point to the significant changes induced by the variance of surface antigens in bacterial cells. The result of this PCA is consistent with SERS spectra presented in Fig. 3a, which show in these areas intensive SERS bands because of the vibrational C-C stretching in phenylalanine (1004 cm^-1^), the C-N stretching mode of the adenine part of flavin adenine dinucleotide (730 cm^-1^), amide III (1280 cm^-1^), and CH_2_ deformation (1400 and 1450 cm^-1^).

All these SERS bands originate mainly from the components of the bacterial cell wall and membrane. The tentative assignments of the recorded SERS bands of *L. monocytogenes* are presented in Table S2 (see ESM). For example, the band at 730 cm^-1^ corresponds to an in-plane ring breathing mode of adenine or from other adenine-bearing molecules, e.g., flavin adenine dinucleotide (FAD), nicotinamide adenine dinucleotide (NAD). The last two molecules are important in the process of cellular respiration taking place in the bacterial cell membrane and demonstrate a close interaction between SERS-active platform and cell wall/ membrane of bacteria. It should likewise be noticed that metabolites of purine degradation may also contribute to the intensity of the band at 730 cm^-1^ [42]. However, according to Premasiri et al. the band at 1352 cm^-1^ (not observed in our spectra) represents a metabolic by-product like pyocyanin, a pigment that bacteria produce during their growth. This suggests little to no interference from environmental contaminations and bacterial metabolic products in our recorded spectra. It should be clarified that bands corresponding to these contaminations may also be masked by strong bacterial signals and therefore not affect the discrimination analysis. Moreover, the bands at 1004, 1278, 1400, and 1450 cm^-1^ correspond to protein vibrational modes and contribute to highest weight in PCA differentiation. This suggests that the spectral differences between the examined strains come mainly from different bacterial envelope proteins.

As genoserotyping is based on differences in surface antigens, it is possible to see the changes in SERS signal during measurements carried out on bacteria strains belonging to different genoserogroups.

**Fig. 4:**
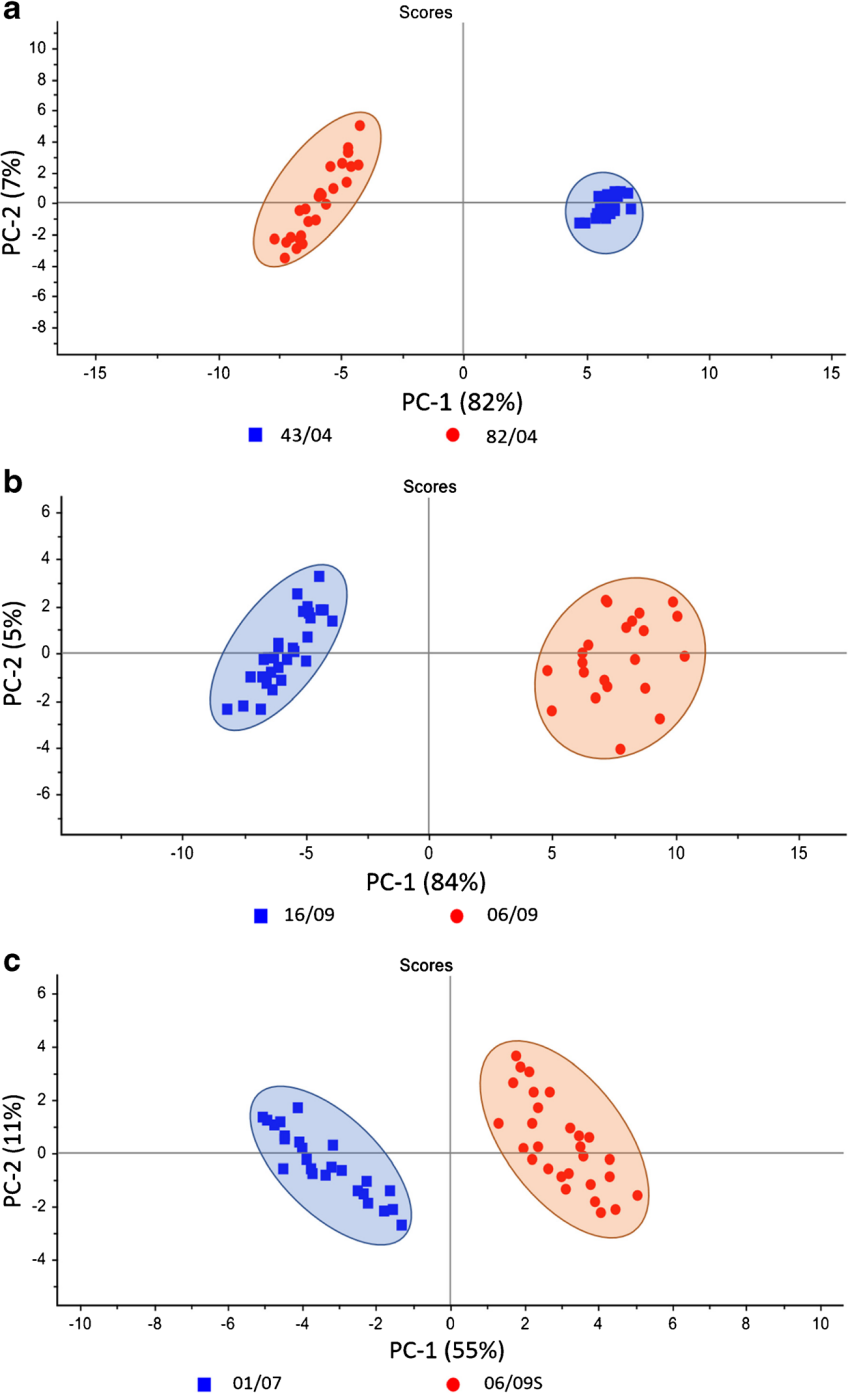
PCA of two *L. monocytogenes* strains containing: (**a**) cadA1 gene (43/04 and 82/04) from different genoserogroups (IIa and IVb); (**b**) cadA2 gene (16/09 and 06/09) from different genoserogroups (IVb and IIc), and (**c**) both *cadA2* and *bcrABC* genes (01/07 and 06/09S) from different genoserogroups (IVb and IIa). For all spectra the excitation wavelength was at 785 nm, laser power at 0.5 mW, and acquisition time 45 s. Each SERS spectrum was averaged from at least 25 measurements at different sites of the SERS

In the next step, the comparison of the SERS spectra of strains belonging to different genoserogroups of *L. monocytogenes*, containing *cadA1* gene (strains 43/04 and 82/04, Fig. 4a), *cadA2* gene (strains 16/09 and 06/09, Fig. 4b), or *cadA2* and *bcrABC* genes (strains 01/07 and 06/09/S, Fig. 4c) was performed. This was done to confirm the results from the previous experiment, which proved the possibility of assigning *L. monocytogenes* strains to specific genoserogroups. Here, the strains with the same set of resistance genes but from different genoserogroups were matched. This means that the only difference between the compared strains was in the types of surface antigens.

As can be observed in Fig. 4a-c, for each pair of *L. monocytogenes* strains the calculated scores are grouped into two clusters. The obtained PC-1 and PC-2 values yield 84% of total variance for two *L. monocytogenes* strains containing *cadA1* gene (43/04 and 82/04) from IIa and IVb genoserogroups, respectively (Fig. 4a). For the two strains within two genoserogroups both with *cadA2* gene, 06/09 (gr IIc) and 16/09 (gr IVb), the calculated PC-1 and PC-2 values were also high and explained the 89% of the total variance among the studied samples (Fig. 4b). PCA also revealed differentiation between two strains within two genoserogroups both with *cadA2* and *bcrABC* genes, 06/09/S (gr IIa) and 01/07 (gr IVb) with 66% accuracy.

These data strongly indicate the possibility of classification among strains from different genoserogroups. This means that the SERS technique is able to distinguish the surface antigen profile of *L. monocytogenes* bacteria from one genoserogroup from bacteria representing other genoserogroups. In many cases the information derived from the cell envelope is sufficient to determine the genoserogroup and strain of a bacterium. Our experiments prove that the SERS signals of bacterial cells come mainly from the cell envelope and molecules located in close proximity to it.

Moreover, we wanted to check whether it is possible to detect SERS spectra changes caused by the presence of the proteins determining resistance to heavy metal (Cd^2+^) and disinfectant (BC). In some bacterial strains there are specific genes whose expression is manifested by the presence of efflux pumps. Such pumps can extrude one definite substrate or a range of compounds out of the bacterial cell, leading to drug resistance [43]. This may refer to quaternary ammonium compounds such as benzalkonium chloride (BC), which are extensively used in the food processing environment and also in health care [44]. One of the known mechanisms leading to BC resistance in *L. monocytogenes* is connected with the presence of the resistance cassette *bcrABC*, which is composed of TetR family transcriptional regulator (*bcrA*) and two SMR genes (*bcrB* and *bcrC*). SMR protein is a proton-depended multidrug efflux system. This efflux system was first identified on a large, ca 80 kb, plasmid pLM80 [32].

So far it has been demonstrated that the pLM80 plasmid plays a role not only in conferring BC resistance on particular *L. monocytogenes* strains, but also in the cadmium (Cd^2+^) resistance of these strains because of the presence of the *cadA2* gene [31]. The other cadmium resistance determinant, *cadA1*, was harbored on the Tn*5422* transposon, associated with plasmids of various sizes [45]. The CadA1 and CadA2, which are the Cd^2+^-ATPases, belong to the Zn^2+^/Cd^2+^/Pb^2+^-ATPase bacterial subfamily of P_1B_-ATPases that ensure detoxification of bacteria [46]. The efflux pump encoded by *cadA1* and *cadA2* genes helps bacteria to avoid poisoning by Cd^2+^ and Zn^2+^. Interestingly, it has been shown that Cd^2+^-resistant isolates that were also resistant to BC were more likely to harbor *cadA2* alone or together with *cadA1* than isolates that were Cd^*2+*^ resistant but BC susceptible [31].

The sizes of the mentioned efflux pumps are different. CadA1 is composed of 711 amino acids (aa), CadA2 of 705 aa, while BcrB of 106 aa and BcrC of 113 aa. As can be easily calculated, the CadA proteins are more than three times larger than the BC efflux pump system; however, their main parts are located in the cytoplasm. The CadA proteins are composed of eight transmembrane helices. In turn, each SMR protein (BcrB and BcrC) is composed of four transmembrane α-helices [47], giving a final efflux pump composed of eight transmembrane helices. This means that the sizes of the transmembrane parts of these two pumps are more or less the same. This information seems to be crucial for observing these proteins in SERS spectrum with the same probability.

**Fig. 5:**
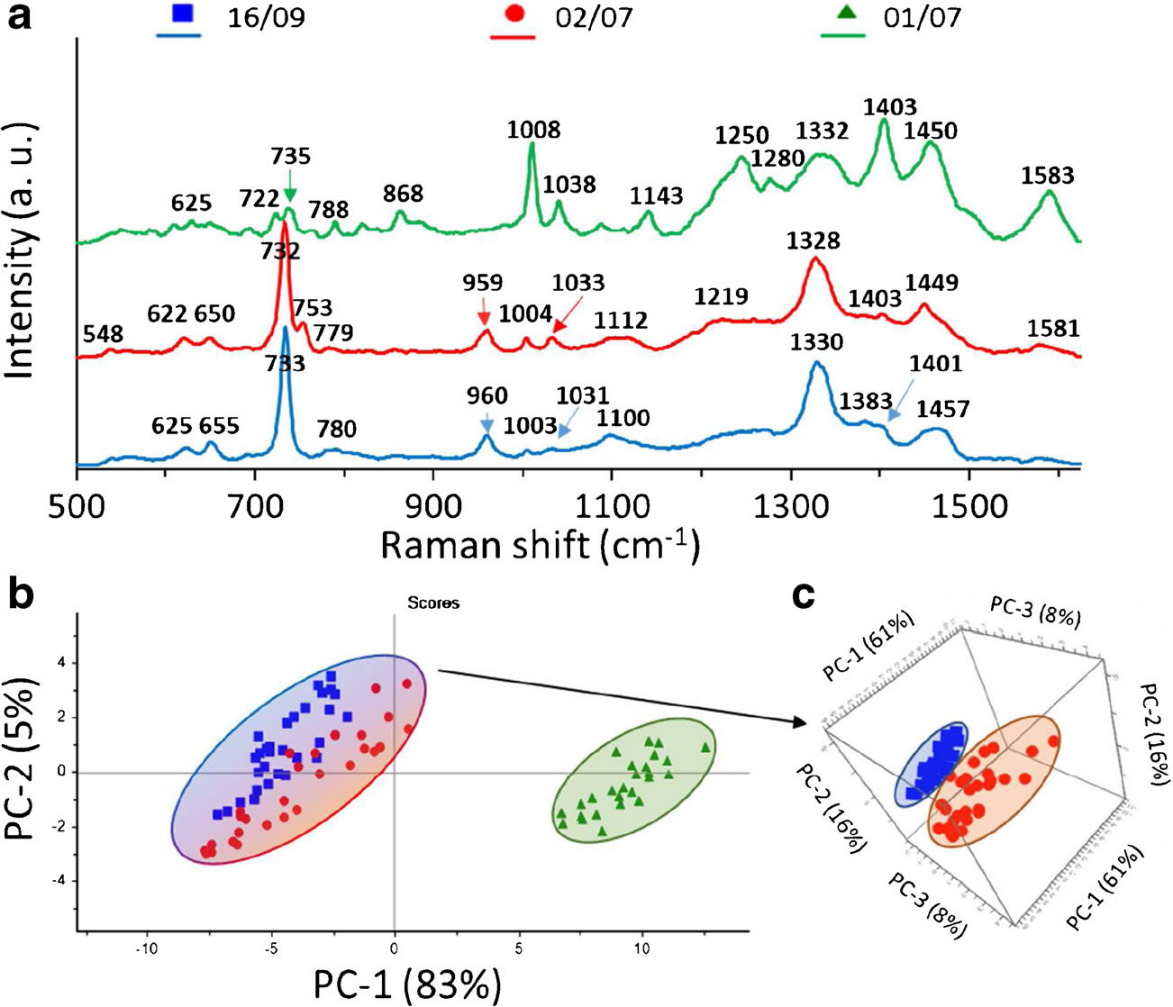
Average SERS spectra (**a**) and PCA (**b**) of three *L monocytogenes* strains from one genoserogroup (IVb) filtered from saline solution and recorded on Ag:Au SERS platforms; PCA of two *L monocytogenes* strains: 16/09 and 02/07 (**c**). For all spectra, the excitation wavelength was at 785 nm, laser power at 0.5 mW, and acquisition time 45 s. Each SERS spectrum was averaged from at least 25 measurements at different points of the SERS platform

In this work, we compared the control strain 02/07 with a strain containing *cadA2* gene (16/09) and a strain containing both *cadA2* and *bcrABC* genes (01/07), all from the same genoserogroup (IVb). It is clear that it is possible to distinguish, with high accuracy, a strain containing *bcrABC* cassette from strains without it. The average SERS spectra of three *L. monocytogenes* strains from one genoserogroup (IVb) exhibit some differences (Fig. 5a). The highest changes can be seen for strain 01/07, in which one can observe a strong decrease in the intensity of the band at ~730 cm^-1^ and increased intensities of the bands at 1008, 1250, 1403, and 1583 cm^-1^. Strains 16/09 and 02/07 have very similar SERS spectra, but they exhibit a key difference in the band at 753 cm^-1^, which is absent in the case of strain 16/09 and present in the case of strain 02/07.

As seen in Fig. 5b, the calculated PC-1 and PC-2 values yield 88% of total variance for these two *L. monocytogenes* strains with the *bcrABC* cassette (red and blue marks) and without it (green marks) and illustrate their excellent differentiation. For better visualization of differentiation, especially between 16/09 and 02/07 samples, the PCA scores are presented in 3D (Fig. 5c; ESM Figs. S5, S6).

**Fig. 6:**
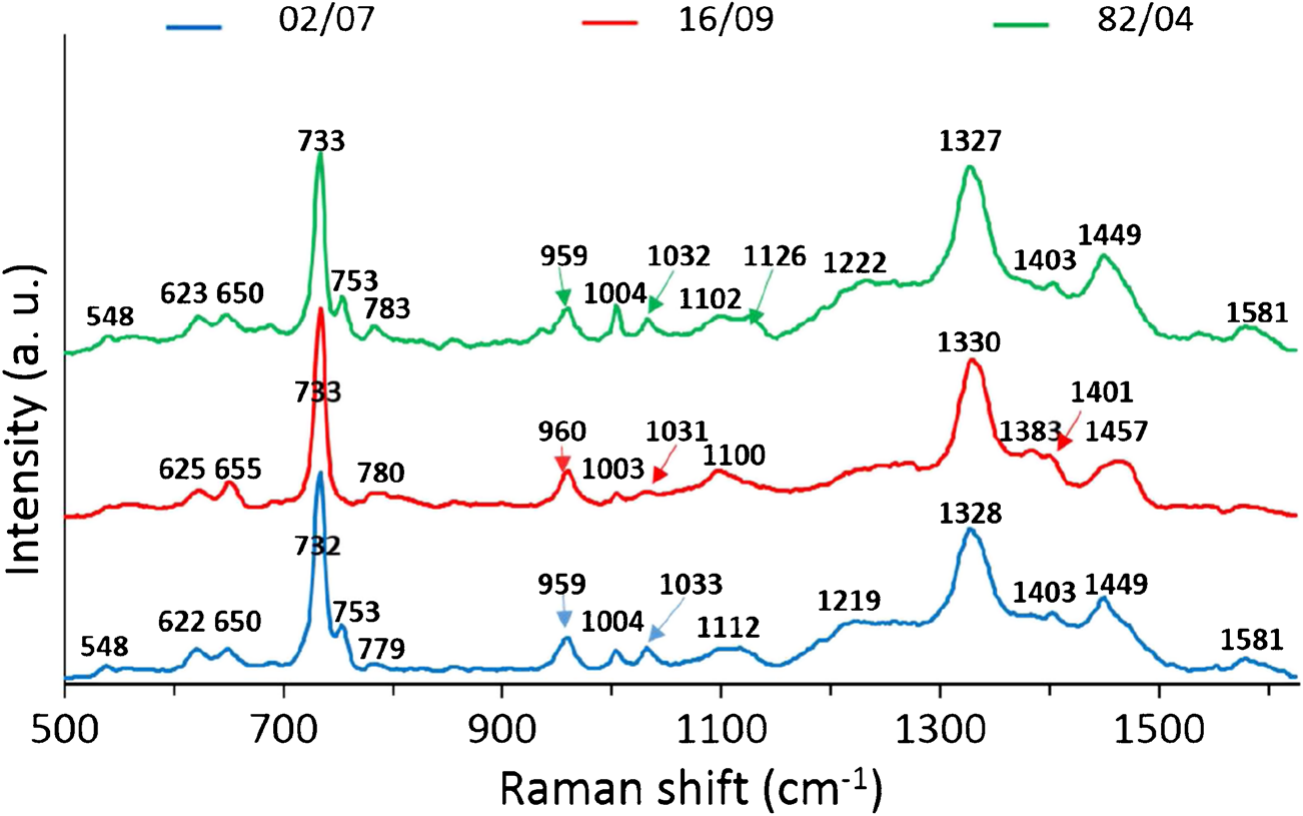
The average SERS spectra of three *L. monocytogenes* strains (16/09, 02/0, and 82/04) from one genoserogroup (IVb). For all measurements excitation wavelength was at 785 nm, laser power at 0.5 mW, and acquisition time was 45 s. Each SERS spectrum was averaged from at least 25 measurements at different points of the SERS platform

In the next step, we have checked whether the presence of *cadA* determinants (without *bcrABC* cassette) affects the SERS spectrum of bacteria. The control strain (02/07) with strains containing *cadA1* (82/04) or *cadA2* genes (16/09) have been compared. All strains where from the same genoserogroup (IVb). As one can notice in Fig. 6, the SERS spectra of all studied bacteria look very much alike. The only difference was the small peak located at ~753 cm^-1^, which was present in the case of strains 02/07 and 82/04 and absent in the case of the strain 16/09. For this reason it was not possible to distinguish these strains with high accuracy via PCA (ESM Fig. S7).

**Fig. 7:**
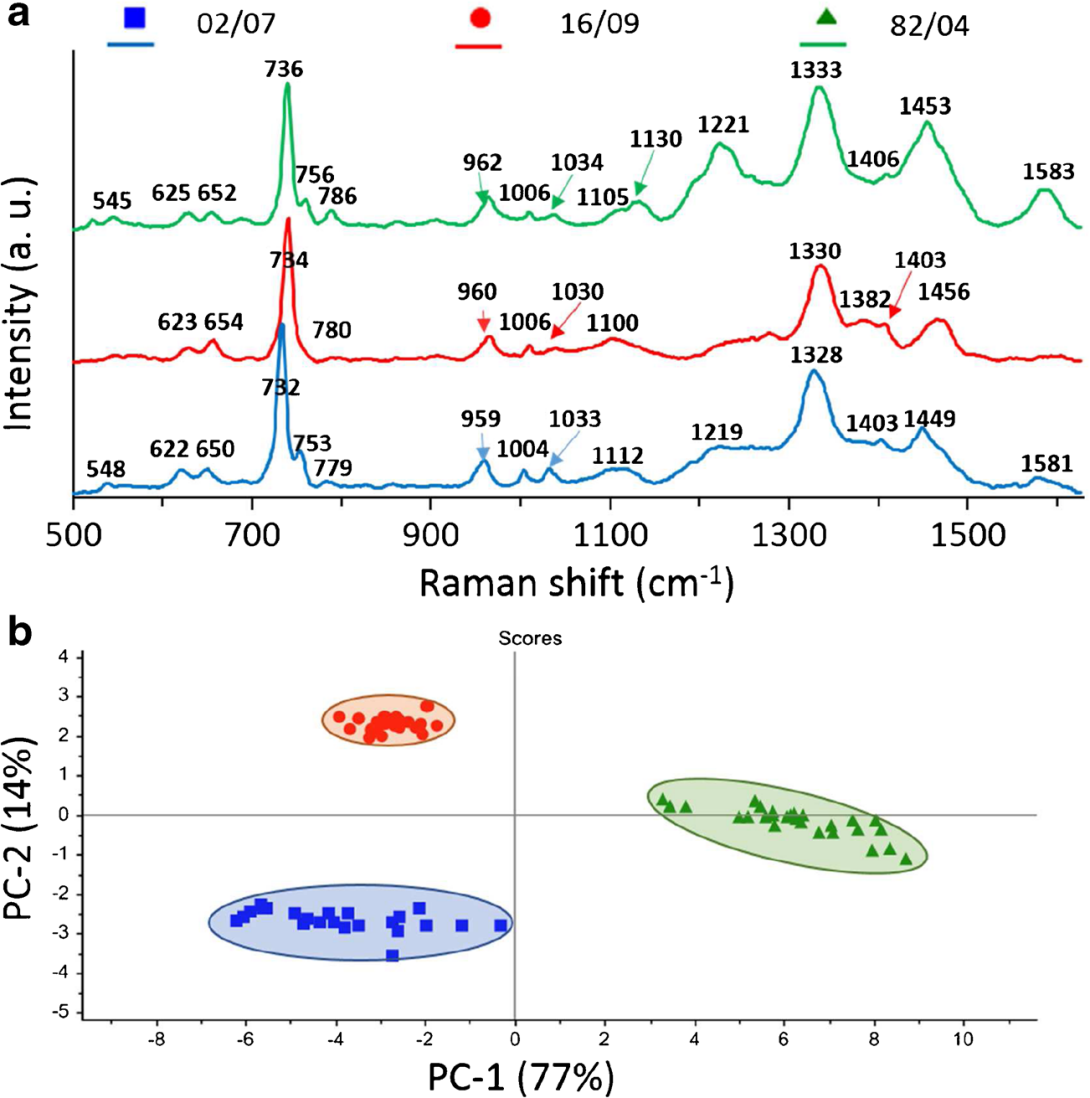
The average SERS spectra (**a**) and PCA (**b**) of three *L. monocytogenes* strains (16/09, 02/07, and 82/04) from one genoserogroup (IVb). Strains 16/09 and 82/04 were cultured on medium supplemented with CdCl_2_. For all measurements excitation wavelength was at 785 nm, laser power at 0.5 mW, and acquisition time was 45 s. Each SERS spectrum was averaged from at least 25 measurements at different places of the SERS platform

Finally, the same strains as discussed above (16/09, 02/07, and 82/04) were applied in new experimental conditions in order to check whether the *cadA* genes expression would be seen as the change of the SERS spectrum. To perform this approach, the strains 16/09 and 82/04 were grown on MH+HB broth supplemented with 75 μg/mL anhydrous cadmium chloride (Fig. 2). The obtained data were matched with 02/07 strain grown on MH+HB broth without supplementation, as it is sensitive to Cd^2+^ and will not grow on medium supplemented with cadmium chloride. As can be seen in Fig 7a, the averaged SERS spectra of three *L. monocytogenes* strains look remarkably different. The difference between the strains 02/07 and 16/09 in the form of the absence/presence of the band 753 cm^-1^ can still be seen. However, the spectrum of the strain 82/04 has changed a lot – we can see increased bands intensities at ~1221 cm, 1453, and 1583 cm^-1^. The band at 1221 cm^-1^ is associated with proteins in cell membrane and also with cytosolic proteins [48]. The band at 1453 cm^-1^ also originates from protein bands (umbrella mode of methoxyl [4] and C-H bending mode of structural proteins [49]). The bacteria bands at 753 cm^-1^ (symmetric breathing of tryptophan [50]) and 1583 cm^-1^ (C=C bending mode of phenylalanine [51]) originate from tryptophan and phenylalanine, respectively, two crucial amino acids in the bacterial proteins. This suggests that the observed spectral differences between analyzed strains come mainly from the different bacterial surface proteins.

The resulting PC-1 versus PC-2 scores calculated for the described SERS data give 91% of total variance (PC-1 plus PC-2). This demonstrates excellent differentiation of these strains with high accuracy. Figure 7b presents three separated clusters corresponding to the strains 16/09 (red), 82/04 (green), and 02/07 (blue) from one genoserogroup (IVb) of *L. monocytogenes.*

It should be highlighted that in Figs. 5 and 7 the obtained scores (in percentage values) of PC1 and PC2 are very similar and give together 88% and 91% variance, respectively. The main difference concerns the cluster/inter-score distances, which are larger in Fig. 7 and indicate a better distinction among studied samples.

Moreover, the validation of PCA method used for identification of *L. monocytogenes* at strain level for three food matrices was performed. In the first step, the PCA analysis for three strains, 16/09 (red), 82/04 (green), and 02/07 (blue) from one genoserogroup (IVb) of *L. monocytogenes* from selected food medium (three food samples were studied and a total 75 SERS spectra were collected; 25 SERS spectra for all studied strains) was applied to build the PCA model. Then the additional data of the test sample [external food sample (smoked salmon) with known strain identified by PCR method] were introduced into this model. The calculated PCA scores are included in Fig. S8 (see ESM) as asterisks. The test sample is located in the clusters of the model PC scores corresponding to particular strain of analyzed species. These results present the analytical potential of SERS technique combined with PCA analysis towards strain level bacteria identification.

In summary, this work demonstrates the possibility of detecting BC efflux pump in *L. monocytogenes* cell membrane and sheds new light on the opportunity offered by SERS technology in bacteria identification at the strain level. However, the selectivity manifested by the possibility of observing BC efflux pumps and the lack of opportunity to observe CadA proteins in bacteria grown on MH+HB without supplementation was somehow disturbing. Such results, however, could be well explained. It turns out that in spite of genomic proximity of *cadA2* and *bcrABC* genes on pLM80, the cassettes mediating resistance to BC and to Cd^2+^ are not regulated by the same substrates. As proven previously by Elhanafi et al. in 2010, after the exposure of strains containing *bcrABC* cassette, increased *bcrABC* transcript levels are observed; however, these transcripts are also detected even in the absence of the disinfectant [32].

Interestingly, such results were not observed for *cadA* genes. Their expression is induced only in the presence of Cd^2+^ Zn^2+^ Pb^2+^ Bi^3+^, from which Cd^2+^ is the most powerful inducer [52]. This is connected with the fact that Cd^2+^-ATPase from *L. monocytogenes* can use Cd^2+^ as a co-substrate [53]. For this reason, the contribution of CadA proteins was seen in the SERS spectrum only in the case when bacteria were grown on medium supplemented with cadmium chloride (compare Figs. 6 and 7).

Previously, it was shown that the dominant molecular species giving their contribution to the SERS spectra of bacteria excited at 785 nm are the metabolites of purine degradation [42]. However, slight changes of the dominant SERS bands may be caused by the presence of the additional proteins/enzymes produced by bacterial cells, which can be concluded from the SERS experiments performed with *E. coli* strains. Premasiri et al. claimed that the SERS spectrum of *E. coli* 6757, which has the most different drug resistance profile from the other five measured *E. coli* strains, has also the most different SERS spectrum [17].

In the present study, some of the investigated bacterial strains were resistant to BC and cadmium or cadmium alone. The resistance mechanisms were associated with the presence of additional proteins in their cell membrane. Previous experiments showing the possibility of SERS-based bacteria identification did not consider what the molecular resistance mechanism of strains under study was. Hypothetically, this could be the reason for the inability of differentiation of all bacteria strains [17].

## Conclusions

Identification of bacterial strains is very important, especially in the case of disease diagnosis. This in particular applies to strains of bacteria that have acquired resistance to specific compounds, e.g., antibiotics, heavy metals, or disinfectants. The expression of such resistance genes allows the survival of a strain. This article shows that the differentiation between genoserogroups from one bacterial species, *L. monocytogenes*, by SERS-PCA-based experiment is possible, as the detection of proteins determining resistance to heavy metal (Cd^2+^) and disinfectant (BC). A comparison of the obtained PCA results (the sum of PC1 and PC2) is shown in Table S3 (see ESM).

The results shown in this paper indicate that the signal observed during SERS experiments of bacteria cells comes mainly from the cell envelope and molecules located in its close proximity. As long as the expression of some genes is manifested by the presence of specific proteins in the cell membrane and as long as the genoserotyping is based on differences in surface antigens, the changes in SERS signal during measurements carried out on different bacterial strains belonging to a single species will probably be observed. These results demonstrate the rapid identification of bacteria at strain level based on different kinds of surface antigens and resistance genes. The research presented here should open a new path in microbiological diagnostics. In summary, the proposed SERS-PCA-based method of bacteria identification is sensitive, label-free, cost effective, fast, and may be performed using portable Raman equipment. In the future it can become an alternative and robust tool for the identification of pathogens.

## Electronic supplementary material

ESM 1 (PDF 923 kb)
