# Supplementary material for: Strain-level typing and identification of bacteria – a novel approach for SERS active plasmonic nanostructures
Source: Anal Bioanal Chem. 2018 Jun 16;410(20):5019–31. doi: 10.1007/s00216-018-1153-0 (PMC6061775; doi:10.1007/s00216-018-1153-0)
Supplement: Supplementary file 1 — (PDF 923 kb) [file 216_2018_1153_MOESM1_ESM.pdf]

Analytical and Bioanalytical Chemistry

Electronic Supplementary Material

Strain-level typing and identification of bacteria – a novel approach for SERS active plasmonic nanostructures

Evelin Witkowska, Dorota Korsak, Aneta Kowalska, Anna Janeczek, Agnieszka Kamińska

Contents

1. **Primers used for PCR and results of PCR for all studied *Listeria monocytogesnes* strains.**
2. **Capturing substrate characterization.**
3. **Principal component analysis – supplementary results.**

1. Primers used for PCR and results of PCR for all studied *Listeria monocytogesnes* strains

Table S1 Primers used for PCR reactions

Gene targeted	primer	Sequnce (5'-3')	Reference
prfA	Lip1<br>Lip2	GAT ACA GAA ACA TCG GTT GGC<br>GTG TAA TCT TGA TGC CAT CAG G	1
prs	PRS-1<br>PRS-2	GCTGAAGAG ATTGCG AAA GAA G<br>CAA AGA AAC CTT GGA TTT GCG G	2
lmo0737	Lmo0737-1<br>Lmo0737-2	AGG GCT TCA AGG ACT TAC CC<br>ACG ATT TCT GCT TGC CAT TC	2
lmo1118	Lmo1118-1<br>Lmo1118-2	AGG GGT CTT AAA TCC TGG AA<br>CGG CTT GTT CGG CAT ACT TA	2
orf2819	ORF2819-1<br>ORF2819-2	AGC AAA ATG CCA AAA CTC GT<br>CAT CAC TAA AGC CTC CCA TTG	2
orf2110-1	ORF2110-1<br>ORF2110-2	AGT GGA CAA TTG ATT GGT GAA<br>CAT CCA TCC CTT ACT TTG GAC	2
flaA	flaA-F<br>flaA-R	TTA CTA GAT CAA ACT GCT CC<br>AAG AAA AGC CCC TCG TCC	3
cadA1	cadA-Tn5422F<br>cadA-Tn5422R	CAG AGC ACT TTA CTG ACC ATC AAT CGT T<br>CTT CTT CAT TTA ACG TTC CAG CAA AAA	4
cadA2	cadA-pLM80F<br>cadA-pLM80R	ACA AGT TAG ATC AAA AGA GTC TTT TAT T<br>ATC TTC TTC ATT TAG TGT TCC TGC AAA T	4
bcrABC	p1<br>p2	CAT TAG AAG CAG TCG CAA AGC A<br>GTT TTC GTG TCA GCA GAT CTT TGA	5

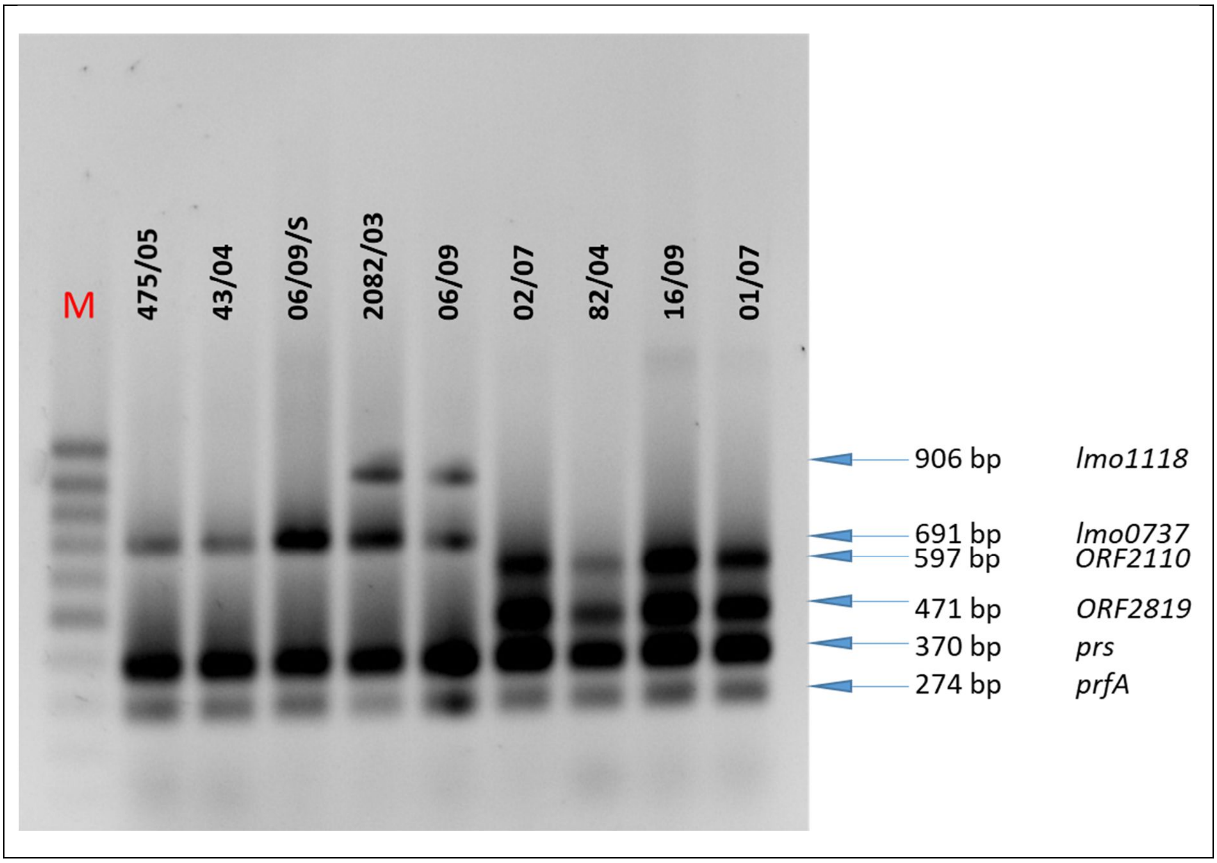

Fig. S1 PCR patterns of molecular *L. monocytogenes* serogroups obtained after agarose gel electrophoresis of DNA products generated by multiplex PCR. Lane 1–3 show amplified fragments for serogroup IIa, lanes 4-5 for serogroup IIc, and lanes 6-9 for serogroup IVb; lane M shows DNA molecular weight marker (GeneRuler 100 bp, DNA Ladder, Fermentas)

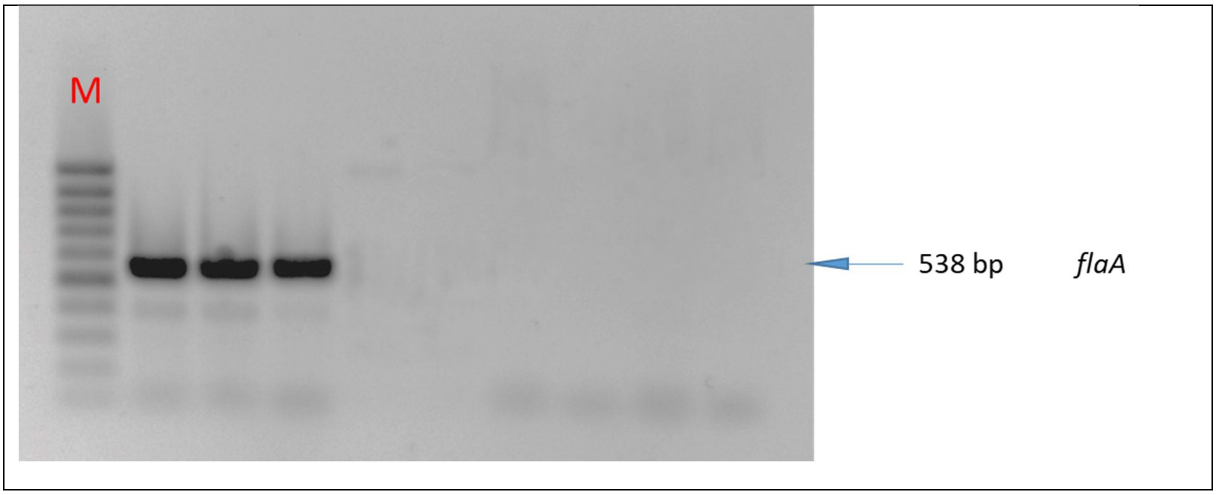

Fig. S2 Lanes 1-3 show positive *flaA* PCR pattern obtained after agarose gel electrophoresis of DNA products generated by duplex PCR; lanes 4-9 show negative *flaA* PCR pattern obtained after agarose gel electrophoresis of DNA products generated by duplex PCR; lane M shows DNA molecular weight marker (GeneRuler 100 bp, DNA Ladder, Fermentas).

2. Capturing substrate characterization

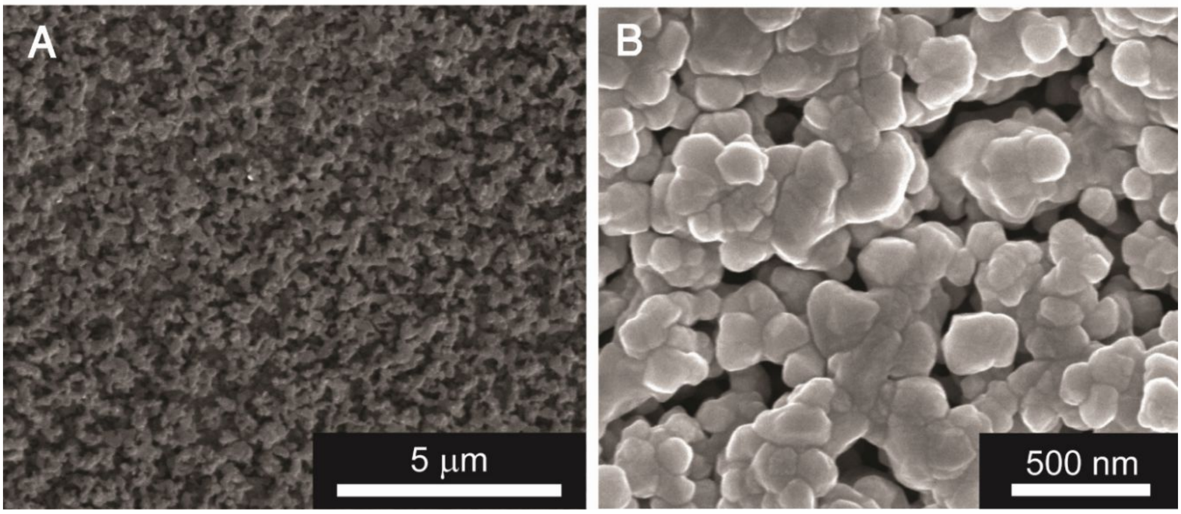

Fig. S3 The SEM images of Ag-Au hybrid surfaces at (A) lower and (B) higher magnifications

Table S2 Band assignment of SERS spectra of *L. monocytogenes*

Assignment	Range
C-O-C ring deformation	540-575
Guanine, tyrosine	640-675
Adenine, glycoside	713-740
Cytosine, uracil	745-790
Symmetric breathing of tryptophan (protein assignment)	752-757
O-P-O (RNA)	800-815
C=C deformation, C-N stretching	930-990
Phenylalanine, C-C aromatic ring stretching	1000-1010
C-C stretching (phospholipids carbohydrates),C-N stretching	1025-1060
O-P-O (DNA), C-C or C-O-C stretching (carbohydrates)	1080-1105
=C-O-C= (unsaturated fatty acids in lipids)	1130-1145
C-O ring, aromatic aminoacids in proteins	1150-1185
Amide III (random), thymine	1215-1295
Amide III (protein), C-H deformation	1315-1325
Adenine, guanine, CH deformation	1330-1345
COO- symmetric stretching	1390-1415
CH_2 deformation of proteins, umbrella mode of methoxyl (4)	1440-1475
Amide II	1510-1560
Adenine, guanine (ring stretching), tryptophan	1570-1595

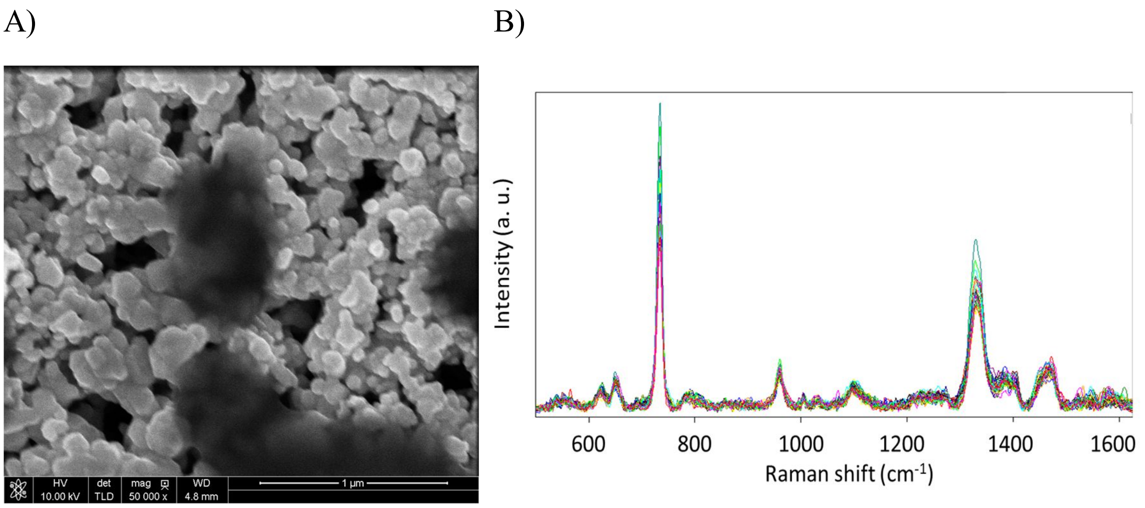

Fig. S4 A) The SEM images of Ag-Au hybrid surfaces covered with single bacteria cells, B) the reproducibility of SERS spectra of *L. monocytogenes* (16/09) recorded in mapping mode within 20 x 24 µm area of SERS-active substrate

3. Principal component analysis – supplementary results

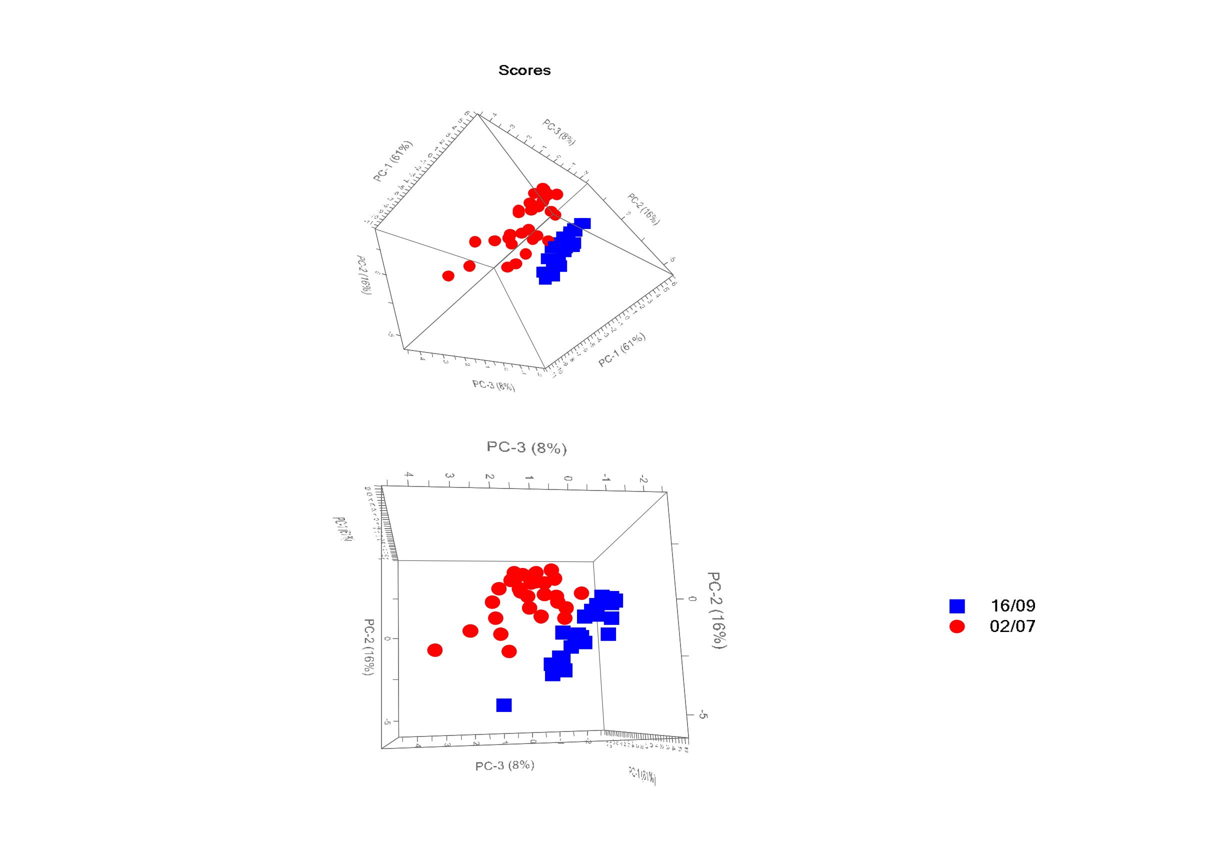

Fig. S5 Data taken form Fig. 5 for direct comparison of both 16/09 and 02/07 samples

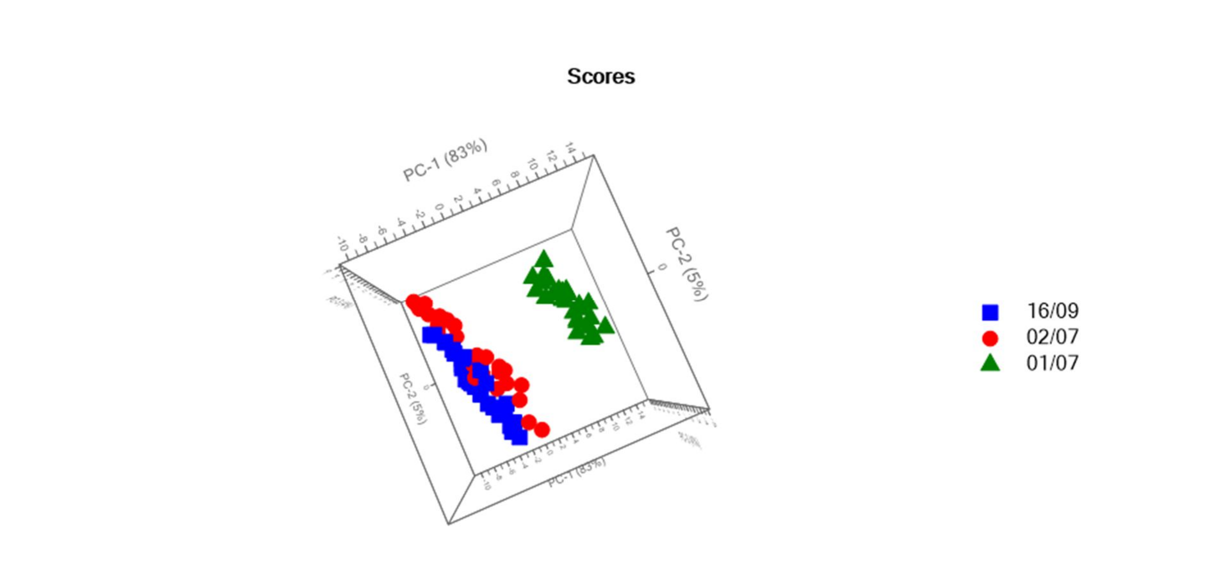

Fig. S6 Data taken from Fig.5 presented in 3D for better visualization

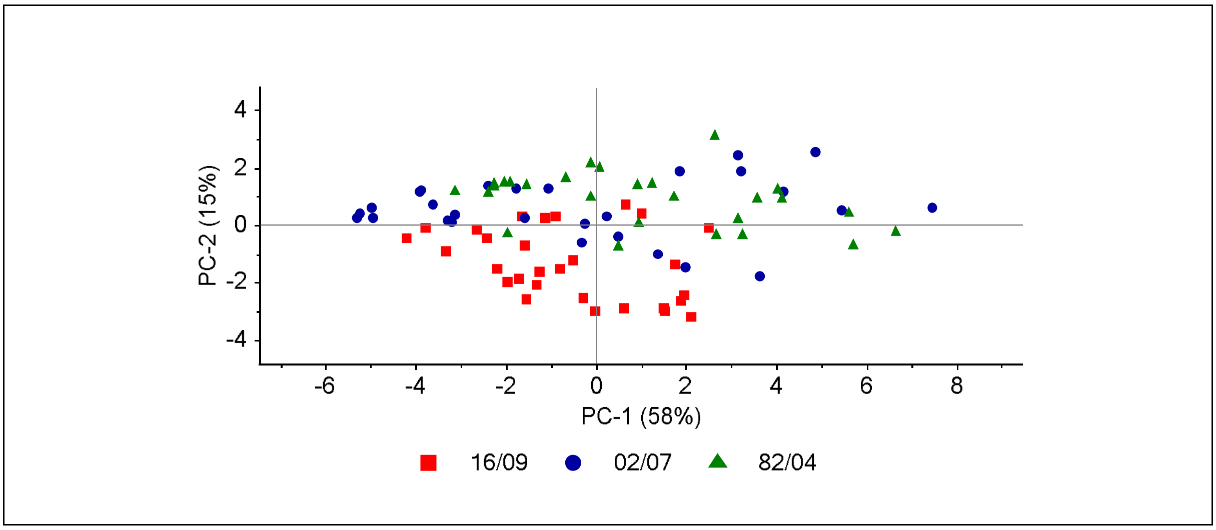

Fig. S7 PCA of three *L. monocytogenes* strains (16/09, 02/07 and 82/04) from one genoserogroup (IVb) cultured on MH+HB medium without supplementation of $CdCl_2$

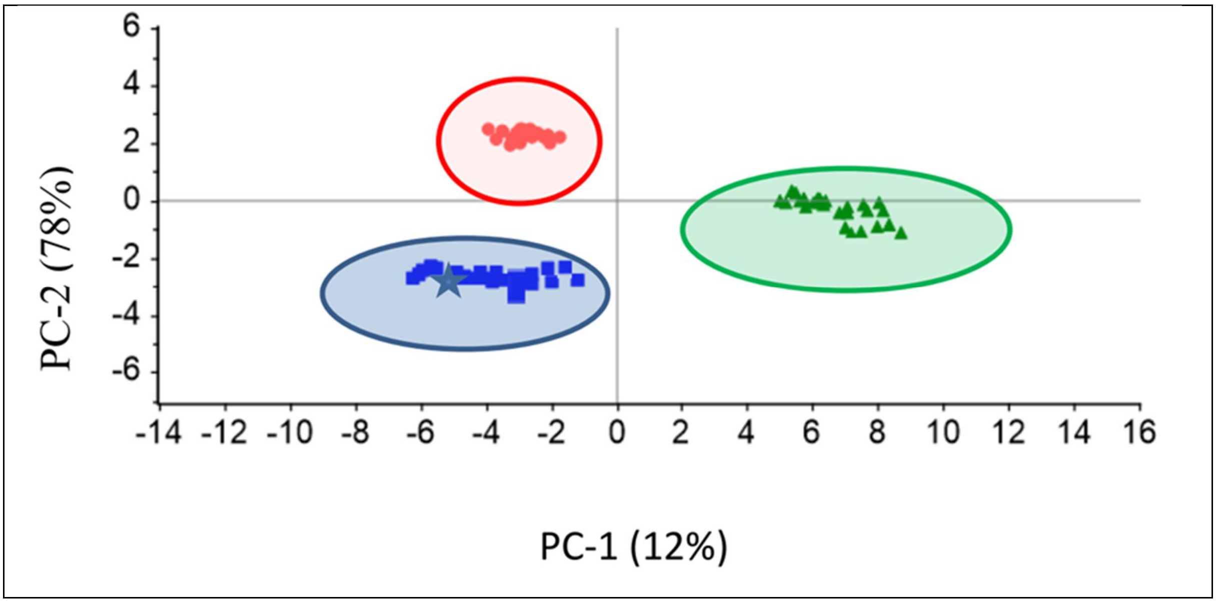

Fig. S8 Scoreplote of PC-1 versus PC-2 component of three *L. monocytogenes* strains : 16/09 (red), 82/04 (green) and 02/07 (blue) from one genoserogroup (IVb). Strains 16/09 and 82/04 were cultured on medium supplemented with $CdCl_2$. Asterisk represents the score calculated for test sample (smoked salmon)

Table S3 The sum of PC-1 and PC-2 calculated for studied strains

**Groups of strains**	**PC1 + PC2 [%]**
Three strains (02/07, 2082/03, 475/05) from different genoserogroups (IVb, IIc, and IIa, respectively)	**92**
Two strains harboring *cadA1* gene (43/04 and 82/04) from different genoserogroups (IIa and IVb, respectively)	**89**
Two strains harboring *cadA2* gene (16/09 and 06/09) from different genoserogroups (IVb and IIc, respectively)	**89**
Two strains harboring *cadA2* and *bcrABC* genes (01/07 and 06/09S) from different genoserogroups (IVb and IIa, respectively)	**66**
Three strains (02/07, 16/09, and 01/07) from one genoserogroup (IVb) harboring different set of genes (control strain, strain with *cadA2* gene, and strain with *cadA2* and *bcrABC* genes, respectively).	**88**
Three strains (02/07, 16/09, and 82/04) from one genoserogroup (IVb) harboring different set of genes (control strain, strain with *cadA2* gene, and strain with *cadA1* gene, respectively).	**91**

REFERENCES

(1) D'Agostino, M.; Wagner, M.; Vazquez-Boland, J. A.; Kuchta, T.; Karpiskova, R.; Hoorfar, J.; Novella, S.; Scortti, M.; Ellison, J.; Murray, A.; Fernandes, I.; Kuhn, M.; Pazlarova, J.; Heuvelink, A.; Cook, N., A Validated PCR-Based Method to Detect *Listeria monocytogenes* Using Raw Milk as a Food Model--Towards an International Standard. *J. Food Prot.* **2004,** *67* (8), 1646-1655.

(2) Doumith, M.; Buchrieser, C.; Glaser, P.; Jacquet, C.; Martin, P., Differentiation of the Major *Listeria monocytogenes* Serovars by Multiplex PCR. *J. Clin. Microbiol.* **2004,** *42* (8), 3819-3822.

(3) Borucki, M. K.; Call, D. R., Listeria monocytogenes Serotype Identification by PCR. *J. Clin. Microbiol.* **2003,** *41* (12), 5537-5540.

(4) Mullapudi, S.; Siletzky, R. M.; Kathariou, S., Diverse Cadmium Resistance Determinants in *Listeria monocytogenes* Isolates from the Turkey Processing Plant Environment. *Appl. Environ. Microbiol.* **2010,** *76* (2), 627-630.

(5) Elhanafi, D.; Dutta, V.; Kathariou, S., Genetic Characterization of Plasmid-Associated Benzalkonium Chloride Resistance Determinants in a *Listeria monocytogenes* Strain from the 1998-1999 Outbreak. *Appl. Environ. Microbiol.* **2010,** *76* (24), 8231-8238.
